# Use of the RETeval™ Handheld Electroretinogram Device for Assessing the Risk of Diabetic Retinopathy in Patients With Type 1 Diabetes: A Case Report

**DOI:** 10.7759/cureus.97206

**Published:** 2025-11-19

**Authors:** Sebastian Sirek, Barbara Trepka-Sirek, Sebastian Seget, Aleksandra Górska, Przemysława Jarosz-Chobot, Dorota Pojda-Wilczek

**Affiliations:** 1 Department of Ophthalmology, Faculty of Medical Sciences in Katowice, Medical University of Silesia, Katowice, POL; 2 Department of Cranio-Maxillo-Facial Surgery, Faculty of Medical Sciences in Zabrze, Medical University of Silesia, Katowice, POL; 3 Department of Pediatrics and Children's Diabetology, Medical University of Silesia, Katowice, POL

**Keywords:** diabetic retinopathy, electroretinography, reteval, retevaltm, type 1 diabetes

## Abstract

Diabetic retinopathy (DR) is the most common and severe ocular complication in diabetes. Significant advances in retinal electrophysiology have emerged in recent years in relation to diabetes-associated eye disease. Growing evidence of neuronal degeneration in DR has been established, most of which comes from electroretinography (ERG) studies.

A five-year-old male patient with type 1 diabetes mellitus (T1D), under treatment since 2021, and a 15-year-old male patient with T1D, under treatment since 2019, underwent ERG testing using the RETeval™ device (LKC Technologies, Inc., Germantown, MD, USA) and the DR program. Hemoglobin A1c (HbA1c) levels were measured at regular intervals at the outpatient clinic. The patients obtained DR protocol scores of 21.4 and 26.0, respectively. The median HbA1c during the study period was 6.2% for the first patient and 8.2% for the second.

The findings suggest that RETeval could have potential clinical utility in the assessment of visual function and the early detection of diabetic eye changes.

## Introduction

Diabetic retinopathy (DR), one of the most significant microvascular complications of diabetes mellitus, contributes substantially to vision impairment among adults worldwide. It affects approximately one-third of individuals with diabetes [1] and represents one of the most prevalent and severe ocular manifestations of the disease. After 20 years of diabetes duration, nearly all patients with type 1 diabetes (T1D) develop some degree of DR. Even during the subclinical stage, functional and microvascular changes can be detected in the retina, including a reduction in the thickness of the inner retinal layers (IRLs) [2]. Chronic hyperglycemia and related metabolic pathways contribute to neurodegeneration, early microvascular damage, and dysfunction of the neurovascular unit. Recent advances in retinal electrophysiology have enhanced understanding of diabetes-related retinal neurodegeneration, much of which has been elucidated through electroretinography (ERG) studies. RETeval is a modern, handheld, non-invasive, full-field flash ERG recording system compliant with the international ISCEV (International Society for Clinical Electrophysiology of Vision) standards [3-5]. In addition, it allows for extended protocol testing, one of which is the DR protocol [5].

## Case presentation

**Figure 1:**
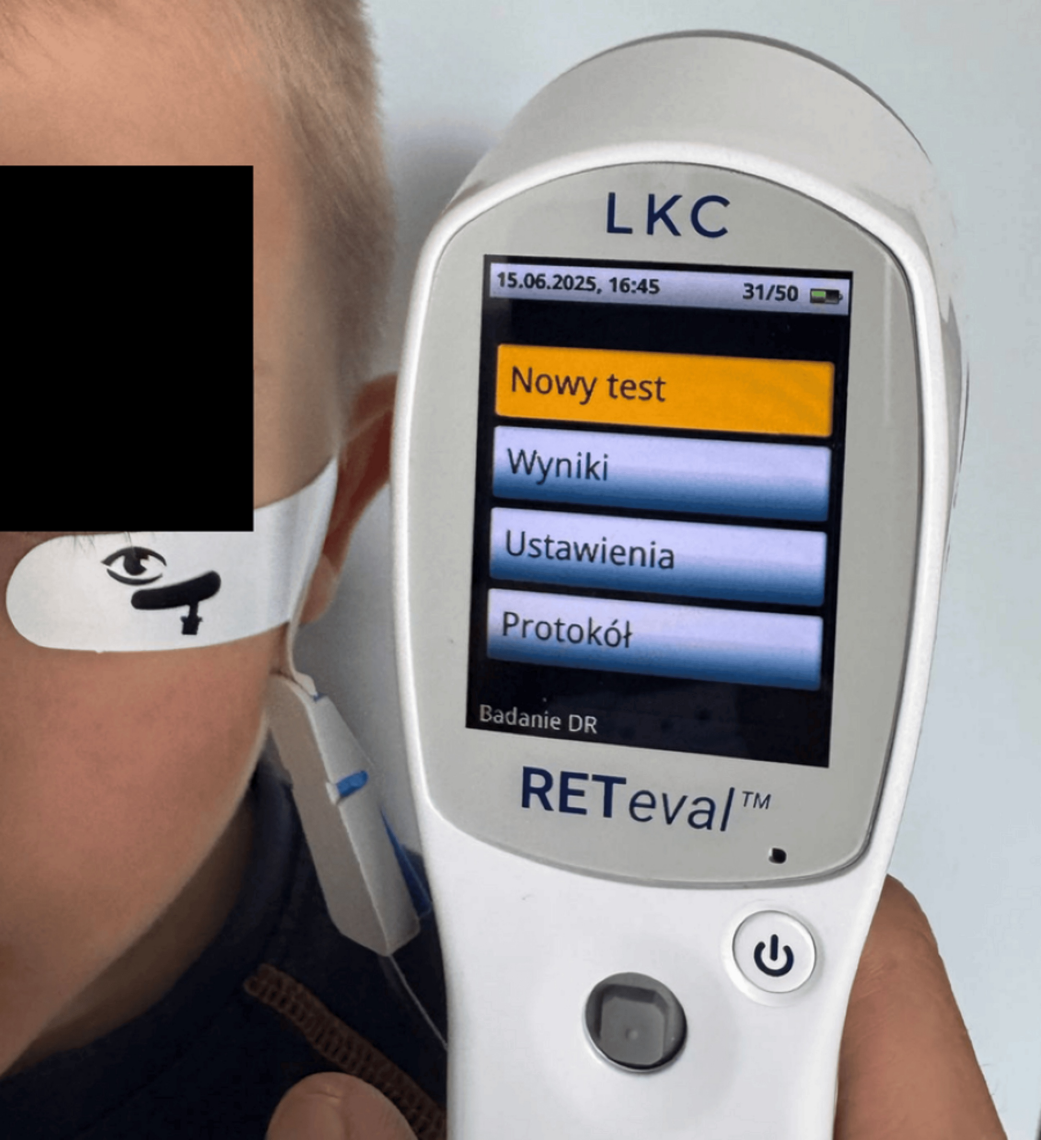
Skin electrode array placed 2 mm below the lower eyelid margin, with the RETeval device

Full-field flash ERG testing was performed using the RETeval™ device (LKC Technologies, Inc., Germantown, MD, USA). A skin electrode array (Sensor Strip, LKC Technologies, Inc.) was applied 2 mm below the lower eyelid margin. The array consists of three electrodes - active (positive), reference (negative), and ground - integrated into a single adhesive strip. Figure 1 illustrates the electrode placement and the positioning of the RETeval device during testing. Flash stimuli were delivered via a 60 mm diameter Ganzfeld dome. Built-in dynamic pupillometry enabled pupil size compensation, allowing testing without pharmacological dilation. The total ERG recording time was approximately 60 seconds for daytime-adapted flash ERG. The DR score integrates ERG and pupillary responses from both eyes into a single numerical indicator of DR. HbA1c levels were measured periodically during follow-up. Both patients were treated with the MiniMed™ 780G (Medtronic, Northridge, CA, USA) insulin pump, operating in the advanced hybrid closed-loop (AHCL) mode. Case 1 has been on therapy since January 2021, and Case 2 since December 2019. No other relevant risk factors were identified in either patient.

Case 1

**Figure 2:**
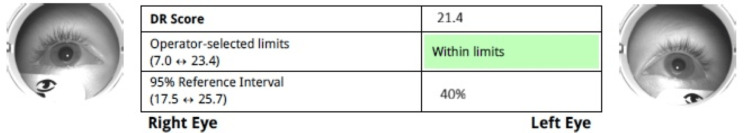
RETeval test protocol: DR examination in a five-year-old patient with T1D DR, diabetic retinopathy; T1D, type 1 diabetes mellitus

**Figure 3:**
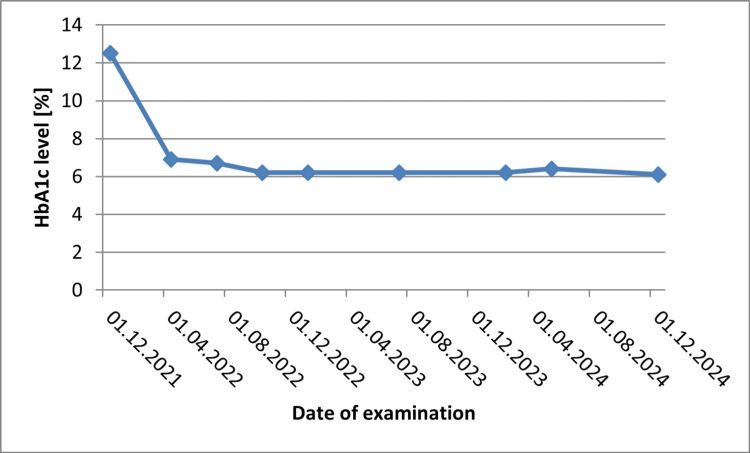
HbA1c level for a five-year-old patient diagnosed with type 1 diabetes in 2021 HbA1c, hemoglobin A1c

A five-year-old boy with a four-year history of T1D achieved a DR protocol score of 21.4 on the RETeval device, within the normal reference range (the examination was conducted on January 6, 2025) (Figure 2). The median hemoglobin A1c (HbA1c) during the study period was 6.2%, as summarized in Figure 3, which illustrates the temporal trend of HbA1c values throughout the monitoring period.

Case 2

**Figure 4:**
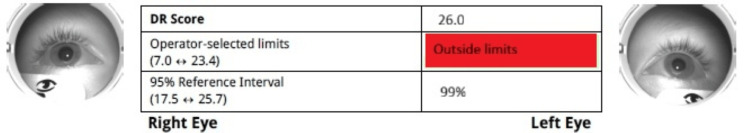
RETeval test protocol: DR examination in a 15-year-old patient with T1D DR, diabetic retinopathy; T1D, type 1 diabetes mellitus

**Figure 5:**
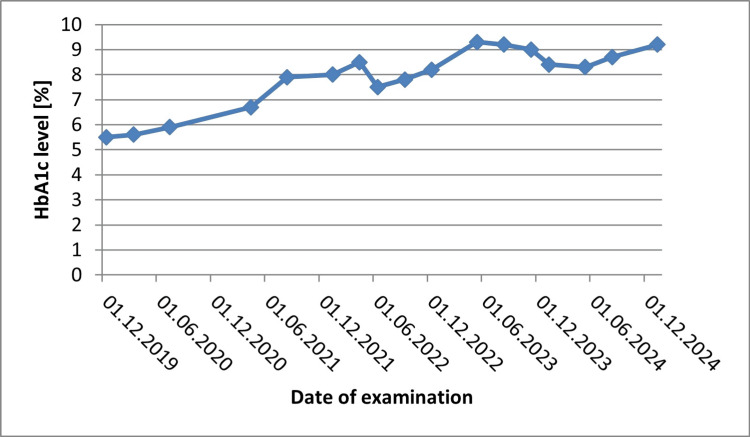
Temporal trends of HbA1c are shown for a 15-year-old patient diagnosed with type 1 diabetes in 2019 HbA1c, hemoglobin A1c

A 15-year-old boy with a six-year history of T1D obtained a DR protocol score of 26.0 (the examination was conducted on March 28, 2025), exceeding the upper normal limit and indicating an increased risk of developing DR. Figure 4 presents the DR score output from the RETeval device for this patient. The median HbA1c during the study period was 8.2%, as shown in Figure 5, which illustrates the changes in HbA1c levels over the observation period.

In both cases, fundus examinations were entirely normal, showing no clinical signs of DR. ERG testing was therefore not performed for diagnostic purposes, but rather to illustrate the potential role of ERG-based assessment in the functional monitoring of retinal health in pediatric patients with T1D.

## Discussion

T1D predominantly affects children, adolescents, and young adults. Standard management involves lifelong subcutaneous insulin therapy, delivered through syringes, insulin pens, or insulin pumps. The AHCL system represents a new era in the treatment of T1D. It is another step toward improving the quality of life, effectiveness, and safety of insulin therapy in people with diabetes. These systems allow for automatic adjustment of the insulin dose to the current needs of the diabetic, using a continuous glucose monitoring (CGM) system, even from the onset of T1D.

This has been shown to result in fewer episodes of hyperglycemia, an increase in the percentage of time spent in the desired glycemic range, improved HbA1c values, and a reduced risk of hypoglycemia. CGM and advanced insulin delivery systems have been associated with improved glycemic control in T1D; however, this study focuses specifically on retinal function assessment using ERG. The HbA1c value indicates the average glycemia over an estimated three-month period, while nearly 50% of it reflects glucose levels from the most recent month. The recommended HbA1c target for patients with T1D is up to 6.5% (48 mmol/mol) [6-8].

The DR RETeval protocol is a test based on standard ISCEV protocols for daytime adaptation and extended protocols, including those developed for patients with diabetes [5,9]. Although skin electrodes were historically avoided in ERG testing because they produce weaker signals, improvements in hardware, data acquisition, and Fourier analysis techniques have enabled the consistent results reported in this study, removing the necessity for corneal electrodes. RETeval calculates a DR risk assessment score by first measuring the default time and amplitude of 16 Td-s and 32 Td-s flashes, along with the pupil area ratio between 4 Td-s and 32 Td-s flashes. It then integrates the implicit time (the interval between the stimulus and the peak of the electrical response), the interpeak amplitude, the patient’s age, and the subject’s pupil response. The normal range for the DR protocol, as set by the operator, is between 7.0 and 23.4 [5,9]. As DR severity progresses, both the velocity and amplitude of pupillary responses decrease, likely reflecting early autonomic neuropathy and impaired retinal function. Smith SA and Smith SE reported that DR protocol scores increased with advancing DR stage, characterized by prolonged implicit times, reduced amplitudes, and impaired pupillary responses [9,10].

The current cases demonstrate that higher HbA1c levels were associated with elevated DR protocol scores. This observation may suggest that worsening glycemic control is associated with functional retinal impairment, even before fundus changes become apparent. In both cases, fundus examination revealed no signs of DR, and no other co-morbidities were identified. However, the DR protocol scores obtained via RETeval demonstrated functional differences in retinal responses, which paralleled differences in glycemic control: Case 1 had a median HbA1c of 6.2%, while Case 2 had a higher median HbA1c of 8.2%. These findings suggest that ERG can detect early functional alterations reflecting the underlying pathophysiology of diabetes, even before structural retinal changes become apparent. Nevertheless, its current clinical applicability remains limited, and ERG should, at present, be considered primarily a research tool rather than a routine diagnostic measure. The observed differences in DR protocol scores between the two cases cannot be attributed solely to age or diabetes duration. Rather, these findings suggest that worsening glycemic control is associated with functional retinal impairment, detectable even before structural fundus changes become apparent. It is necessary to perform the test on a larger study group in order to standardize the results obtained. Attempting to determine the HbA1c threshold at which the first ERG abnormalities are observed using the RETeval device may be a helpful tool in setting individual glycemic control goals and reducing eye complications in diabetes.

A recent study showed that the RETeval DR score, a composite of ERG and pupillary responses, is the strongest predictor of progression to vision‑threatening DR, with scores ≥ 26.9 indicating high risk [11]. In our cases, both patients had normal fundus findings, but the DR protocol scores differed in parallel with glycemic control (median HbA1c, 6.2% vs. 8.2%), which may suggest that functional retinal impairment can precede structural changes. These findings support the potential use of ERG-based assessment for early detection of functional retinal alterations and risk stratification in patients with T1D.

Notably, greater differences between patients were observed in retinal response parameters rather than in pupillary reactivity. In patients with longer disease duration, ERG revealed significant retinal dysfunction, including delayed peak times and reduced wave amplitudes. Regular ERG testing in patients with normal fundus findings and visual acuity may serve as a sensitive measure of subclinical retinal dysfunction, potentially reflecting the early stages of diabetic eye disease.

The RETeval system uses skin electrodes rather than corneal electrodes, which generally produce weaker electrophysiological signals. However, modern signal amplification and advanced processing algorithms in the RETeval device largely compensate for this difference, allowing for reliable detection of retinal responses.

The RETeval DR protocol provides an objective, quantitative score derived from flicker ERG responses and pupil reaction analysis. The score correlates with the severity of DR, as defined by established clinical grading systems such as the Early Treatment Diabetic Retinopathy Study (ETDRS) scale. Higher RETeval scores are associated with increasing DR severity, while lower scores typically correspond to normal or subclinical retinal function [12].

No comparison with conventional full-field ERG or structural imaging modalities, such as fundus photography or optical coherence tomography (OCT), was performed in the present cases. This absence of direct comparison represents a limitation and precludes definitive conclusions regarding the relative diagnostic performance of the RETeval system. Additionally, as these observations are based on only two patients, their generalizability is inherently limited. Despite these constraints, the cases illustrate the feasibility and potential clinical value of RETeval for early functional assessment of the diabetic retina. These findings underscore the need for larger, prospective studies to validate RETeval as a functional monitoring tool. Future research should include comparisons with conventional ERG and structural imaging to clarify the relationship between electrophysiological changes and clinically detectable diabetic retinal abnormalities. Such studies will be essential for determining the role of RETeval in routine clinical practice and its potential for early detection and risk stratification in T1D.

## Conclusions

Over the past two decades, ERG studies have substantially contributed to understanding retinal neurodegeneration in diabetes. Parallel developments in continuous insulin therapy and retinal diagnostics now offer new opportunities for preserving visual function in diabetic patients. Due to its simplicity, noninvasiveness, and standardized scoring, the RETeval DR protocol may serve as a valuable research tool for exploratory functional assessment of the retina in pediatric patients with T1D. In our cases, differences in HbA1c levels were reflected in RETeval DR scores, which may suggest that glycemic control could influence early retinal function. Notably, ERG detected subtle functional alterations despite normal fundus examinations, illustrating its ability to capture early, potentially subclinical changes as part of the diabetic pathophysiological process. However, its current clinical applicability remains limited, and ERG should, at present, be regarded primarily as a research instrument rather than a routine diagnostic tool in patient care.
